# Immune Recognition of the 60kD Heat Shock Protein: Implications for Subsequent Fertility

**DOI:** 10.1155/S1064744996000336

**Published:** 1996

**Authors:** Steven S. Witkin, Jan Jeremias, Andreas Neuer, Sami David, Isaac Kligman, Miklos Toth, Emily Willner, Keren Witkin

**Affiliations:** Department of Obstetrics and Gynecology Cornell University Medical College 515 East 71st Street New York New York 10021 USA

## Abstract

The 60kD heat shock protein (hsp60) is a highly conserved protein and a dominant antigen of most pathogenic bacteria. In some women, chronic or repeated upper genital tract infections with *Chlamydia trachomatis*, and possibly with other microorganisms, induces immune sensitization to epitopes of hsp60 that are present in both the microbial and human hsp60. Once a woman becomes sensitized to these conserved epitpes, any subsequent induction of human or bacterial hsp60 expression will reactivate hsp60-sensitized lymphocytes and initiate a pro-inflammatory immune response. Hsp60 is expressed during the early stages of pregnancy, by both the embryo and the maternal decidua. We examined, therefore, whether women who were sensitized to hsp60 experienced less successful pregnancy outcomes compared to women who were not sensitized to this antigen. In women undergoing in vitro fertilization (IVF), the presence of cervical IgA antibodies reactive with the *C. trachomatis* hsp60 correlated with implantation failure after embryo transfer. Further analysis revealed that an immunodominant epitope for these IgA antibodies was an hsp60 epitope shared between *C. trachomatis* and man. In subsequent studies of women not undergoing IVF, cervical IgA antibodies to the human hsp60 were identified in 13 of 91 reproductive age women. This antibody was most prevalent in those women with a history of primary infertility (*p* = 0.003). In addition, cervical anti-hsp60 IgA correlated with the detection of the pro-inflammatory cytokines interferon-γ (*p* = 0.001) and tumor necrosis factor-α (*p* = 0.02) in the cervix. Conversely, women with proven fertility had the highest prevalence of the anti-inflammatory cytokine, interleukin 10, in their cervices (*p* = 0.001). In an analysis of serum samples in a third study, women with a history of two or more consecutive first trimester spontaneous abortions had a higher prevalence (*p* = 0.01) of IgG antibodies to the human hsp60 (36.8%) than did age matched fertile women (11.1%) or women with primary infertility (11.8%). Immune sensitization to epitopes expressed by the human hsp60 may reduce the probability of a successful pregnancy outcome due to reactivation of hsp60-reactive lymphocytes, induction of a pro-inflammatory cytokine response and interference with early embryo development and/or implantation.


Infectious Diseases in Obstetrics and Gynecology 4:152–158 (1996)
© 1996 Wiley-Liss, Inc.

### Immune Recognition of the 60kD Heat Shock Protein: Implications for Subsequent Fertility

**Steven S. Witkin, Jan Jeremias, Andreas Neuer, Sami David, Isaac Kligman, Miklos Toth, Emily Willner, and Keren Witkin**

*Departments of Obstetrics and Gynecology, Cornell University Medical College and Mount Sinai Medical Center, New York, New York*

### Abstract

The 60kD heat shock protein (hsp60) is a highly conserved protein and a dominant antigen of most pathogenic bacteria. In some women, chronic or repeated upper genital tract infections with *Chlamydia trachomatis*, and possibly with other microorganisms, induces immune sensitization to epitopes of hsp60 that are present in both the microbial and human hsp60. Once a woman becomes sensitized to these conserved epitopes, any subsequent induction of human or bacterial hsp60 expression will reactivate hsp60-sensitized lymphocytes and initiate a pro-inflammatory immune response. Hsp60 is expressed during the early stages of pregnancy, by both the embryo and the maternal decidua. We examined, therefore, whether women who were sensitized to hsp60 experienced less successful pregnancy outcomes compared to women who were not sensitized to this antigen. In women undergoing in vitro fertilization (IVF), the presence of cervical IgA antibodies reactive with the *C. trachomatis* hsp60 correlated with implantation failure after embryo transfer. Further analysis revealed that an immunodominant epitope for these IgA antibodies was an hsp60 epitope shared between *C. trachomatis* and man. In subsequent studies of women not undergoing IVF, cervical IgA antibodies to the human hsp60 were identified in 13 of 91 reproductive age women. This antibody was most prevalent in those women with a history of primary infertility ($p = 0.003$). In addition, cervical anti-hsp60 IgA correlated with the detection of the pro-inflammatory cytokines interferon-$\gamma$ ($p = 0.001$) and tumor necrosis factor-$\alpha$ ($p = 0.02$) in the cervix. Conversely, women with proven fertility had the highest prevalence of the anti-inflammatory cytokine, interleukin 10, in their cervices ($p = 0.001$). In an analysis of serum samples in a third study, women with a history of two or more consecutive first trimester spontaneous abortions had a higher prevalence ($p = 0.01$) of IgG antibodies to the human hsp60 (36.8%) than did age matched fertile women (11.1%) or women with primary infertility (11.8%). Immune sensitization to epitopes expressed by the human hsp60 may reduce the probability of a successful pregnancy outcome due to reactivation of hsp60-reactive lymphocytes, induction of a pro-inflammatory cytokine response and interference with early embryo development and/or implantation. © 1996 Wiley-Liss, Inc.

Key words

*Chlamydia trachomatis*, infertility, spontaneous abortion, 60kD heat shock protein

Most bacteria share a dominant antigenic determinant, designated "common antigen." Subsequent studies[1] revealed that this antigen is a member of the 60kD family of heat shock proteins (hsp60). All other organisms, including mammals, also possess a homologous protein with a related amino acid sequence. Hsp60 functions to prevent the hydrophobic surfaces of unfolded or partially folded nascent proteins from forming nonspecific intracellular aggregates and to promote the proper

Address correspondence to Dr. Steven Witkin, Department of Obstetrics and Gynecology, Cornell University Medical College, 515 East 71st Street, New York, NY 10021.
Supported in part by NIH grant HD 33194 and Deutsche Forschungsgemeinschaft Ne 602/1-1

Received September 15, 1996
Accepted October 1, 1996

Article folding of polypeptide chains. Under conditions of rapid cell growth or differentiation, or following exposure to an environmental stress such as inflammation, fever or toxic chemicals, hsp60 synthesis is increased. By preventing protein denaturation and inappropriate aggregation, the ability of the cell to survive the stress is maximized.[2]

Despite the highly conserved nature throughout evolution of the hsp60 amino acid sequence, bacterial hsp60 is highly immunogenic in man.[3] Typically, following an acute infection immunity is restricted to hsp60 epitopes that are specific to microorganisms. However, prolonged or repeated exposure to bacterial hsp60, such as might occur during an asymptomatic *Chlamydia trachomatis* upper genital tract infection, can trigger immunity to conserved hsp60 epitopes that are also expressed in man.[4–6] Immune sensitization to conserved hsp60 epitopes may facilitate an autoimmune response to self hsp60. The pathogenesis of diabetes in the non-obese diabetic mouse[7] and adjuvant arthritis in rats[8] involve an autoimmune response to hsp60.

We have been exploring the consequences of immune sensitization to hsp60 for fertility in women. Heat shock proteins are among the first proteins produced during embryogenesis,[9] corresponding to a time of rapid development. Epithelial cells in the human decidua also express hsp60.[10] In addition, a murine hybridoma specific for hsp60 has been shown to react with human trophoblast[11] suggesting hsp60-related expression by this tissue. Occlusion of the fallopian tubes subsequent to a chlamydial infection has been attributed to an immune response to the chlamydial hsp60.[4,12] Among women undergoing in vitro fertilization (IVF) the presence of cervical IgA antibodies to chlamydial hsp60 was associated with a failure of implantation after embryo transfer.[13]

Here we present further analyses of the relation between fertility and immune sensitization to the human hsp60.

### MATERIALS AND METHODS

### Subjects

Several different groups of subjects were studied:

#### *Group A.*

This group included 122 women undergoing IVF. Analysis of samples from these women for IgA antibodies to chlamydial hsp60 and *C. trachomatis* structural antigens has previously been reported.[13] IgA antibodies to the chlamydial hsp60 were present in 24 of these women

#### *Group B.*

In this group were 72 women seeking treatment for unexplained infertility, 12 women who had never tried to conceive and 7 women with proven fertility. Among the infertile women, 44 had primary infertility while 28 had at least one child with her present partner. All subjects were married and in monogamous relationship.

#### *Group C.*

This group had 38 women with no live births and a history of at least two consecutive first trimester spontaneous abortions (mean age 37.9 years), 34 female partners of couples with primary infertility of at least three years duration whose husbands had normal semen parameters (mean age 35.7 years) and 36 women with no history of spontaneous abortions who had a live singleton birth within the past three years and who conceived after less than 12 months of attempting to conceive (mean age 35.9 years). The aborter group consisted of 8 women with two abortions, 20 with three abortions, 8 with four abortions and two with greater than four abortions.

### Samples

Endocervical samples were obtained from subjects in group A at the time of oocyte retrieval and from group B at mid-cycle. A Dacron swab was inserted into the endocervix, twirled and removed into a tube containing 0.5 ml phosphate-buffered saline (PBS). The liquid was extruded from the swab, the sample microcentrifuged and the supernatants were frozen at −80°C until used. Serum samples only were obtained from subjects in group C.

### Synthetic Peptide Epitopes of hsp60

Previous studies have demonstrated that sera from women with chlamydial infections recognized 13 peptide epitopes of the chlamydial hsp60.[14] Each of these peptides was synthesized and biotinylated (Chiron Mimotopes, Clayton, Australia) employing a spacer arm at the amino end and an amide at the carboxy terminal. The peptides were reconstituted in 0.2 ml 100% dimethyl sulfoxide, diluted 1:100 in PBS containing 0.1% bovine serum albumin

(BSA) and 0.1% sodium azide and stored in aliquots at −80°C.

### Anti-peptide Antibody Detection

The biotinylated peptides were diluted 1:10 in PBS-0.1% BSA-0.1% sodium azide and 0.1 ml (1.0 μg) added to wells of a microtiter plate containing bound streptavidin. The plate was incubated on a shaker for 60 min at room temperature, and the wells washed four times with PBS-0.1% Tween 20 detergent (PBS-Tween). The cervical samples from group A patients were diluted 1:10 in PBS-2% BSA-0.1% azide and added to the wells. After 120 min at room temperature the wells were washed four times with PBS-Tween and 0.2 ml of a 1:200 dilution of horseradish peroxidase-labelled antibody to human IgA in PBS-2% BSA was added. After a 60 min incubation the wells were washed four times with PBS-Tween followed by two additional washes with PBS. The peroxidase substrate, 2,21-azino-di[3-ethylbenzothiazoline sulphonate] and $H_2O_2$ in 0.1M phosphate/0.08M citrate buffer, pH 4.0 was then added. After 60 min the optical density of each well was determined at 405 nm. Utilizing cervical samples from 98 women previously shown to lack IgA antibodies to intact chlamydial hsp60, a mean optical density value plus two standard deviations of between 0.30 and 0.33 was obtained with each of the peptides. Therefore, a cervical sample was scored as positive for IgA antibody to a synthetic peptide if it yielded an optical density $\geq$ 0.35.

### Antibodies to Human hsp60

Recombinant human hsp60 (StressGen, Victoria, B.C.) was diluted to 10 μg/ml in 0.1M carbonate buffer, pH 9.8 and 0.1 ml added to wells of a microtiter plate. After an overnight incubation at 4°C the wells were washed four times with PBS-Tween. Aliquots (0.1 ml) of cervical samples (group B), diluted 1:5 in PBS-Tween, or sera (group C), diluted 1:160, were added to the wells and the plate floated on a 37°C water bath for 60 min. The wells were then washed and incubated with a 1:500 dilution of alkaline phosphatase (AP)-conjugated goat antibody to human IgA (cervical samples) or a 1:200 dilution of AP-conjugated goat antibody to human IgG (serum samples). Following an additional 37°C 60 min incubation the wells were washed as above and the colorless AP substrate, p-nitrophenylphosphate in 10% diethanolamine buffer was added. After a 30–60 min room temperature incubation the appearance of a yellow color in the wells was quantitated at 405 nm. Known positive and negative samples were always assayed in parallel to the test samples. Inter- and intra-assay variations were <10%. A positive sample was defined as one yielding an optical density value that was at least two standard deviations above the mean value obtained with a panel of samples from subjects of proven fertility and no history of abortions or genital tract infections.

### Cytokine Assays

Cervical samples (group B) were diluted 1:4 and utilized in immunoassays using commercially available ELISA kits (BioSource International, Camarillo, CA) for detection of tumor necrosis factor-α (TNFα), interferon-γ (IFNγ) and interleukin 10 (IL10). Replicate analyses differed by less than 10%.

### Statistics

Fisher's exact test was utilized to examine differences in discrete variables among the groups of subjects. Differences in cervical IgA binding to each of the synthetic peptides were evaluated by the nonparametric Wilcoxon rank sums test. A $p$ value <0.05 was considered significant.

### RESULTS

### Cervical Immunity to a Conserved Epitope of the Chlamydial hsp60

A single immunodominant epitope, comprising amino acids 260–271 of the *C. trachomatis* hsp60, was identified by antibodies present in cervical samples from the IVF subjects (Table 1). More women previously shown to be sensitized to the intact chlamydial hsp60 had cervical IgA antibodies to this peptide than to any of the other peptides. In addition, this peptide yielded the highest mean optical density in the ELISA detection assay ($p < 0.05$).

Cervical immunity to peptide 260–271 was identified in 21 (87.5%) of the 24 women with, and in only 6 (6.1%) of the 91 women without, antibodies to intact hsp60 ($p < 0.0001$). As an immunological marker for detection of cervical IgA antibodies to chlamydial hsp60, peptide 260–271 antibodies had a sensitivity of 87.5%, a specificity of 93.9%, a positive predictive value of 77.8% and a negative predictive value of 96.8%.

**TABLE 1. Cervical IgA antibodies to synthetic peptides corresponding to epitopes of the *C. trachomatis* hsp60 in women undergoing IVF who were positive for antibodies to intact hsp60**

| Amino acid sequence | Amino acid positions | No. Positive (%) | Mean optical density (S.D.) |
|---|---|---|---|
| VTLGPKGRHVVI | 29–40 | 15 (62.5) | .389 (.134) |
| VLAEAIYTEGLR | 94–105 | 12 (50.0) | .359 (.126) |
| SANNDAEIGNLI | 151–162 | 9 (37.5) | .321 (.103) |
| DVVDGMNFNRGY | 188–199 | 16 (66.7) | .389 (.115) |
| SGIKDFLPVLQQ | 228–239 | 14 (58.3) | .358 (.124) |
| ATLVGNRIRGGF | 260–271 | 21 (87.5) | .463 (.175)* |
| GDRRKAMFEDIA | 282–293 | 10 (41.7) | .340 (.118) |
| ILPGGGTALIRC | 411–422 | 14 (58.3) | .381 (.116) |
| NEDEQIGARIVL | 435–446 | 11 (45.8) | .348 (.120) |
| SAPLKQIAANAG | 450–461 | 12 (50.0) | .358 (.131) |
| NAGKEGAIIFQQ | 459–470 | 17 (70.8) | .394 (.152) |
| GAIIFQQVMSRS | 464–475 | 15 (62.5) | .398 (.136) |
| QVMSRSANEGYD | 470–481 | 18 (75.0) | .391 (.150) |

*$P < .05$ vs. any other peptide.

The relation between IVF outcome and cervical immunity to peptide 260–271 is shown in Table 2. Women with this antibody had a significantly ($p$ = 0.03) increased prevalence of transient biochemical pregnancies (22.2%) after embryo transfer than did antibody-negative women (7.4%). Similarly, the presence of anti-peptide 260-271 antibody correlated with a decreased rate of term pregnancy (11.1% vs. 30.5%, $p$ = 0.04). Women positive for this antibody were significantly ($p$ = 0.03) older (37.2 years) than the antibody negative women (34.9 years).

**TABLE 2. Relation between outcome of IVF cycle and cervical antibodies to synthetic peptide 260–271 of the chlamydial hsp60**

| | IgA antibodies to peptide 260–271 Number of patients (%) | |
|---|---|---|
| IVF Outcome | Present | Absent |
| No fertilization | 4 (14.8) | 6 (6.3) |
| Embryo transfer: | | |
| Not pregnant | 12 (44.4) | 48 (50.5) |
| Biochemical pregnancy | 6 (22.2)* | 7 (7.4) |
| Spontaneous abortion | 2 (7.4) | 5 (5.3) |
| Term birth | 3 (11.1)** | 29 (30.5) |

*$p$ = .03 vs. term births; **$p$ = .04 vs. term births.

### Cervical Immunity to the Human hsp60

Among the 91 non-IVF patients whose cervical samples were analyzed, 13 (14.3%) were positive for IgA antibodies to the human hsp60. The presence of this antibody correlated with detection of IFNγ ($p$ = 0.001) and TNFα ($p$ = 0.02) in the cervical samples (Table 3). In contrast, there was no relation between cervical IL10 and anti-hsp60 IgA.

The relation between cervical immunity to the human hsp60 and pregnancy outcome in the subjects studied is shown in Table 4. Women with primary infertility had a significantly ($p$ = 0.003) higher prevalence of cervical anti-hsp60 IgA (10 of 44, 22.7%) than did all of the other women (1 of 47, 2.1%). Among the cytokines, the only significant difference noted ($p$ = 0.001) was a higher prevalence of cervical IL10 among the fertile women (5 of 7, 71.4%) as opposed to women with either primary or secondary infertility (9 of 72, 12.5%).

There was no relation between cervical immunity to hsp60 and time since last sexual intercourse, the presence of cervical antisperm antibodies or detection of cervical *Ureaplasma urealyticum* or *Mycoplasma hominis*.

### Circulating IgG Antibodies to Human hsp60 and Fertility Status

At a serum dilution of 1:160 antibodies to the human hsp60 were more prevalent in women with a history of spontaneous abortions than in the fertile ($p$ = 0.01) or infertile ($p$ = 0.01) women (Table 5). This

TABLE 3. Relation between cytokines and IgA antibodies to the Human 60kD heat shock protein in the cervix

| Pair | No. positive/ Total no. (%) | *p* value |
|---|---|---|
| Hsp60 IgA in women with IFN-γ | 5/9 (55.6%) | 0.001 |
| Hsp60 IgA in women without IFN-γ | 8/91 (8.8%) | |
| Hsp60 IgA in women with TNF-α | 8/31 (25.8%) | 0.02 |
| Hsp60 IgA in women without TNF-α | 5/69 (7.2%) | |
| Hsp60 IgA in women with IL10 | 4/22 (18.2%) | 0.4 |
| Hsp60 IgA in women without IL10 | 9/78 (11.5%) | |

TABLE 4. Relation between IgA antibody to the human 60kD heat shock protein and pregnancy history

| Pregnancy history | No. subjects | No. (%) with anti-heat shock protein IgA |
|---|---|---|
| Primary infertility | 44 | 10 (22.7)*,** |
| Secondary infertility | 28 | 0 |
| Fertile | 7 | 1 (14.3) |
| Conception not attempted | 12 | 0 |

*$p = 0.005$ vs. secondary infertility; **$p = 0.003$ vs. all others.

TABLE 5. IgG antibodies to the 60kD human heat shock protein in sera from females

| Group | No. of women | No (%) positive |
|---|---|---|
| Fertile | 36 | 4 (11.1) |
| Aborters | 38 | 14 (36.8)*,** |
| Infertile | 34 | 4 (11.8) |

*$p = .016$ vs. the infertile group; **$p = .014$ vs. the fertile group.

antibody was present in 14 (36.8%) of the aborters, 4 (11.1%) of the fertile women and 4 (11.8%) of the infertile women. Among the aborters, there was no relation between the number of spontaneous abortions and prevalence of anti-hsp60 IgG.

There was no relation between self-reported history of genital tract infections and serum anti-hsp60 IgG. It should be noted, however, that only 4 women reported a history of *C. trachomatis* infection and none had been infected with *Neisseria gonorrhoeae*. Similarly, there was no relation between past mode of contraception and prevalence of this antibody.

### DISCUSSION

In the present study, the *C. trachomatis* hsp60 epitope comprising amino acids 260–271 was shown to be a dominant epitope recognized by immunocompetent cells in the genital tracts of women undergoing IVF. This agrees with and extends an earlier study identifying the 201–300 amino acid region of the chlamydial hsp60 as an immunodominant epitope in infertile women.[15] Peptide 260–271 is a highly conserved hsp60 epitope; a similar sequence is present in the human hsp60 and antibodies to the chlamydial epitope react with the corresponding human epitope.[14]

It is interesting to speculate on the consequences for women of immune sensitization to hsp60 epitope 260–271. Immunization of rats with an overlapping hsp60 epitope, comprising amino acids 256–269, led to a downregulation of pro-inflammatory immune responses.[16] This suggests that immunity to epitope 260–271 may similarly limit the magnitude of pro-inflammatory immune responses to *C. trachomatis* in the female genital tract.

However, the existence of immune cells that are sensitized to epitope 260–271 of the chlamydial hsp60, and which also recognize the human hsp60, may create complications in those instances where human hsp60 expression occurs. The observed relationships between immune sensitization to epitope 260–271 and both an increased rate of embryo loss after a transient implantation and a decreased pregnancy rate supports the hypothesis that an autoimmune response to the human hsp60 might contribute to adverse pregnancy outcome. Reactivation of hsp60-sensitized lymphocytes by human hsp60 expression in the decidua[10] or embryo[9,11] may interfere with immune regulatory mechanisms essential to the maintenance of the semi-allogeneic embryo and induce immune rejection.

In subsequent studies we identified cervical IgA antibodies to human hsp60 in the cervices of 14.3% of 91 reproductive age women and demonstrated an association between this immune sensitization and both the presence of pro-inflammatory cytokines IFNγ and TNFα in the cervix and a diagnosis of primary infertility.

Cytokines can be classified as enhancing cell-mediated immunity (TH1 responses) or promoting humoral immunity (TH2 responses). Both IFNγ and TNFα are TH1 cytokines while IL10 is a TH2 cytokine which blocks pro-inflammatory cytokine synthesis.[17] Since pro-inflammatory cytokines are generally harmful to pregnancy, and TH2 cytokines predominate at the maternal-fetal interface,[18] it has been hypothesized that maintenance of a TH2 immune response is a prerequisite to a successful pregnancy outcome.[19] Our observation of an association between endocervical IL10 and a history of fertility supports this view and further suggests that TH2 predominance in the cervix may also preferentially occur during the nonpregnant state. Therefore, the observed association between cervical immunity to human hsp60 and IFN$\gamma$ and TNF$\alpha$ expression strongly implies that an immune response to hsp60 may favor TH1 cytokine expression and overcome TH2 cytokine predominance.

Finally, systemic immunity to the human hsp60 was shown to be associated with first trimester spontaneous abortion. Binding of this antibody to hsp60 expressed on maternal or fetal cells, and the subsequent induction of pro-inflammatory cytokines might account for this observation. Either a direct impairment of cell viability or interference with immune regulatory mechanisms could lead to an unfavorable pregnancy outcome.

These observations reinforce the connection between immune sensitization to epitopes of the hsp60 that are expressed in the human hsp60 and adverse pregnancy outcome. Immunity to heat shock proteins has also been observed in a number of autoimmune diseases.[20, 21] Microorganisms such as *C. trachomatis* which induce asymptomatic infections, and which, therefore, tend to be chronic and undetected, are prime candidates as inducers of immunity to crossreacting conserved epitopes of hsp60. Alternatively, immunity to the human hsp60 may also be directly induced by some noninfectious event.

Additional prospective studies are required to clarify the consequences of autoimmunity to the human hsp60 in pregnant women. In addition, there is a need to characterize the cellular locations of hsp60 in maternal and fetal cells during early pregnancy. Such studies are in progress.

### ACKNOWLEDGMENT

The expert technical assistance of Ann Marie Bongiovanni, Vera Yusufu, Alice Godbey and Julius Smalls is gratefully acknowledged.

### REFERENCES

1. Thole JER, Hindersson P, de Bruyn J et al.: Antigenic relatedness of a strongly immunogenic 65kDa mycobacterial protein antigen with a similarly sized ubiquitous bacterial common antigen. Microbial Pathogenesis 4:71–83, 1988.
2. Ellis J: Stress proteins as molecular chaperones. In: van Eden W, Young DB (eds): Stress Proteins in Medicine. New York: Marcel Dekker Inc., pp 1–26, 1996.
3. Kaufmann SHE: Heat shock proteins and the immune response. Immunol Today 11:129–136, 1990.
4. Morrison RP, Manning DS, Caldwell HD: Immunology of *Chlamydia trachomatis* infections. Immunoprotective and immunopathogenetic responses. In: Quinn TC (ed): Sexually Transmitted Diseases. New York: Raven Press, pp 57–84, 1992.
5. Witkin SS, Jeremias J, Toth M, Ledger WJ: Cell-mediated immune response to the recombinant 57-kDa heat-shock protein of *Chlamydia trachomatis* in women with salpingitis. J Infect Dis 167:1379–1383, 1993.
6. Witkin SS, Jeremias J, Toth M, Ledger WJ: Proliferative response to conserved epitopes of the *Chlamydia trachomatis* and human 60-kilodalton heat shock proteins by lymphocytes from women with salpingitis. Am J Obstet Gynecol 171:455–460, 1994.
7. Elias D, Markovits D, Reshef T, Van der Zee R, Cohen IR: Induction and therapy of auto-immune diabetes in the non-obese diabetic (NOD/Lt) mouse by a 65 kDa heat shock protein. Proc Natl Acad Sci USA 87:1576–1580, 1990.
8. Hogervorst JM, Boog CJ, Wagenaar PA, Wauben HM, van der Zee R, van Eden W: T cell reactivity to an epitope of the mycobacterial 65-kDa heat-shock protein (hsp65) corresponds with arthritis susceptibility in rats and is regulated by hsp65-specific cellular responses. Eur J Immunol 21:1289–1296, 1991.
9. Bensuade O, Morange M: Spontaneous high expression of heat-shock proteins in mouse embryonal cells and ectoderm from day 8 mouse embryo. EMBO J 2:173–177, 1983.
10. Mincheva-Nilsson L, Baranov V, Yeung MM, Hammarstrom S, Hammarstrom ML: Immunomorphologic studies of human decidua-associated lymphoid cells in normal early pregnancy. J Immunol 152:2020–2032, 1994.
11. Heybourne K, Fu YX, Nelson A, Farr A, O'Brien R, Born W: Recognition of trophoblasts by $\gamma\delta$ T cells. J Immunol 153:2918– 2926, 1994.
12. Witkin SS: Immune pathogenesis of asymptomatic *Chlamydia trachomatis* infections in the female genital tract. Infect Dis Obstet Gynecol 3:169–174, 1995.
13. Witkin SS, Sultan KM, Neal GS, Jeremias J, Grifo JA, Rosenwaks Z: Unsuspected *Chlamydia trachomatis* infection and in vitro fertilization outcome. Am J Obstet Gynecol 171:1208–1214, 1994.
14. Yi Y, Zhong G, Brunham RC: Continuous B cell epitopes in *Chlamydia trachomatis* heat shock protein 60. Infect Immun 61:1117–1120, 1993.
15. Arno JN, Yuan Y, Cleary RE, Morrison RP: Serologic responses of infertile women to the 60-kD chlamydial heat shock protein (hsp60). Fertil Steril 64:730–735, 1995.
16. Anderton SM, van der Zee R, Noordzij A, van Eden W: Differential mycobacterial 65-kDa heat shock protein T

cell epitope recognition after adjuvant arthritis-inducing or protective immunization protocols. J Immunol 152:3656–3664, 1994.

17. Moore KW, O'Garra A, Malefyt R, Vieira P, Mosman TR: Interleukin 10. Annu Rev Immunol 11:165–190, 1993.
18. Lin H, Mosman TR, Guilbert L, Tuntipopipat S, Wegmann TG: Synthesis of T helper 2-type cytokines at the maternal-fetal interface. J Immunol 151:4562–4573, 1993.
19. Wegmann TG, Lin H, Guilbert L, Mosmann TR: Bidirectional cytokine interactions in the maternal-fetal relationship: is successful pregnancy a TH2 phenomenon. Immunol Today 14:353–356, 1993.
20. Georgopoulos C, McFarland H: Heat shock proteins in multiple sclerosis and other autoimmune diseases. Immunol Today 14:373–375, 1993.
21. Panchapakesan J, Daglis M, Gatenby P: Antibodies to the 65 kDa heat shock proteins in rheumatoid arthritis and systemic lupus erythematosus. Immunol Cell Biol, 70:295–300, 1992.
